# Current Perspectives on the Immunopathogenesis and Pathology of Small Ruminant Lentivirus Infections

**DOI:** 10.3390/v18101165

**Published:** 2026-10-20

**Authors:** Bianca Santana de Cecco, Regina Tose Kemper, Peres Ramos Badial

**Affiliations:** 1 Department of Pathobiology and Population Medicine, College of Veterinary Medicine, Mississippi State University, Starkville, MS 39762, USA; prb132@msstate.edu; 2 School of Veterinary Medicine and Bioscience, SRUC Aberdeen Campus, Craibstone Estate, Aberdeen AB21 9YA, UK; regina.kemper@sruc.ac.uk

**Keywords:** goats, lentivirus, sheep

## Abstract

Small ruminant lentiviruses (SRVLs), including ovine maedi-visna virus and caprine arthritis-encephalitis virus, are considered genetically a single pathogen that causes a progressive multisystemic inflammatory disease. Continuous interspecies transmission in areas where these species come into contact, along with dual infection in individual animals, results in recombination between variants. The main routes of infection are ingestion of infected colostrum and/or milk and inhalation of respiratory secretions. SRLVs primarily target monocytes/macrophages and dendritic cells. Following infection, chronic lymphocytic inflammatory infiltrates are observed in the main target organs: lungs, brain, joints, and mammary gland. Infection with the virus causes lifelong infection in its host, characterized by slow progression until overt disease, which is observed in approximately one-third of the infected animals, occurs. The affected system appears to depend on SRLV strain tropism, the species affected, and the genetic background of each breed or animal. This review examines the immunopathogenesis and pathology of small ruminant lentiviruses.

## 1. Introduction

Small ruminant lentiviruses (SRLVs), which include ovine maedi-visna virus (MVV) and caprine arthritis-encephalitis virus (CAEV), are viruses that cause persistent and progressive chronic diseases in sheep and goats [1]. SRLV infections are worldwide, with prevalence increasing with management intensification [2,3,4].

SRLVs are single-stranded, positive-sense RNA lentiviruses that replicate by producing a provirus, a DNA intermediate that integrates into the host cell genome in both dividing and non-dividing cells [5]. Genetic variability is an important feature of the SRLV genome, and SRLV quasi-species are frequently generated through mutation, recombination, and selection pressure by the host immune system [1]. This mutation and recombination potential implies high genetic and antigenic diversity, with five main genotypes (A–E) and more than 28 subgroups already characterized [1,6]. Genotype A is a diverse group that contains the classic strains of MVV [7,8,9]; genotype B, which has strains that correspond to CAEV [10,11]; genotype C includes SRLV present in Norwegian sheep and goats [12,13]; genotype D has been identified by analyzing *pol* sequences of SRLV of Spanish and Swiss sheep [14]; and genotype E has been isolated in Northern Italy and Sardinia [4,15,16,17,18]. Recent studies in Belgium confirmed SRLV in 13% of sheep farms (A1 subtype) and 17% of goat farms (B1 subtype) [19,20]. Additionally, one of these studies indicated cross-species transmission of SRLV strains between sheep and goats and vice versa, which the authors designated as a new subtype called B5 [20].

Mixed flocks of sheep and goats are epidemiologically important because of cross-species transmission, and multiple factors participate in the formation of new SRLV subtypes [21,22,23]. There is clear evidence of a genetic continuum of caprine and ovine field isolates that do not cluster with the animal species from which they were isolated, and evidence of mixed infections under field conditions, leading to new viral recombinants capable of overcoming species barriers and demonstrating cross-species transmission/infection [24]. Additionally, reports described naturally infected goats with persistent infections with both CAEV and MVV, with detection of viral chimeras generated by recombination between these variants [25]. SRLV variability is important for the virus to escape the host immune response and produce persistent infection. Additionally, variability is likely involved in crossing species barriers [21]. Wildlife species, including red deer (*Capreolus capreolus*), are also susceptible to infection; however, there is no evidence that the virus can replicate or cause disease in these animals [26,27,28,29]. Natural infection with SRLV was also confirmed in Rocky Mountain goats (*Oreamnos americanus*) [30] and Alpine ibexes (*Capra ibex*) [31], while experimental infection has been reported in mouflons (*Ovis aries musimon*) [32], suggesting wild ruminants may serve as reservoirs for SRLVs.

SRLVs negatively impact small ruminant flocks by decreasing welfare and causing economic losses from culling, reduced milk yields, reduced birth weight, and reduced weight gain [21,33,34]. Illius et al. [35] suggest that selectively culling underperforming older ewes can not only reduce disease transmission but also limit ongoing production losses in affected sheep flocks. Currently, no vaccines or effective treatments exist against SRLV, and control measures and management practices remain the only available tools [3,34].

This narrative review integrates relevant published literature on SRLV, with a focus on immunopathogenesis and pathology. The literature discussed was selected based on its relevance to the scope of this review, prioritizing studies that elucidate viral and host factors involved in infection and persistence, innate and adaptive immune responses, and the relationship between these mechanisms and the development of lesions in small ruminants. This review also examines the main clinicopathological manifestations of SRLV infection.

## 2. Immunopathogenesis

SRLV is transmitted directly between adults (aerosols) or through ingestion of milk or colostrum from infected dams [1,33,36,37,38,39]. Horizontal infections are the main route of virus transmission in sheep; however, in goats there is a predominance of lactogenic transmission [38,39,40,41]. Current studies demonstrate that in sheep, maternal transmission alone cannot sustain infection in a flock; therefore, SRLV prevalence cannot increase unless conditions allow horizontal transmission [40,41]. Main risk factors associated with horizontal transmission include prolonged housing periods, animal crowding, and intensive management systems [3,38,39,42]. Recently, animal transit was also identified as a risk factor associated with horizontal transmission of SRLV in caprine herds [36]. Epidemiologic modeling estimates that transmission rates amongst housed sheep are approximately 1000-fold higher than among pastured sheep, in which transmission is considered negligible [3]. Additionally, involved SRLV subtypes are suspected to play an essential role in the efficiency of lactogenic and horizontal transmission [37,38]. Other routes of transmission, such as transplacental and through semen, are less important from an epidemiological point of view [3,5,33].

Sheep and goats respond differently to SRLV infection [43]. In sheep, antibodies in the udder can reduce detection of proviral DNA, but this has not been observed in goats [22,43]. Barquero et al. [22] conducted a 6-month study testing milk from intensively reared ewes and goats. A high percentage of sheep were SRLV-positive by Polymerase Chain Reaction (PCR) performed in milk before antibodies were detected in blood, and most of them became PCR-negative shortly after the first detection of antibodies. The authors suggested that antibodies could have a neutralizing effect. Additionally, an equal percentage of sheep were always PCR-negative in milk, but either became ELISA positive or were always ELISA positive, suggesting that the viral infection occurred in a different location than the udder [22]. In contrast, results in goats did not follow any pattern, and most goats positive by PCR failed to develop antibodies throughout the experiment, hypothesizing that SRLV infection and antibody response in goats and sheep may significantly diverge [22].

It is not clear whether transmission among animals occurs via free virions or through infected cells, including macrophages and epithelial cells [33,43]. Infection occurs across mucosal surfaces, and the main target cells for viral replication are those of the monocyte-macrophage lineage. However, replication occurs only with macrophage maturation in tissue [44,45]. Monocyte differentiation into macrophages increases the expression of various transcription factors, triggering transcription of proviral DNA and leading to the production of new viral particles [34]. Monocytes and immature macrophages are capable of evading the immune system, thus allowing the virus to spread throughout the body [46]. Dendritic cells then migrate to the lymph node, where the virus transfers to macrophages that exit the lymph node, allowing systemic and bone marrow infection [33]. Such viral transfer to macrophages depends on CD4+ T cells [47]. In the bone marrow, the virus may infect myeloid stem cells or stromal cells, followed by proviral integration, allowing the persistent production of infected cells and lifelong infection of the animal [5,48,49]. In addition, other cell types, including mammary gland epithelial cells and microglial cells of the central nervous system, can serve as reservoirs [33]. Unlike other lentiviruses, SRLV levels in the blood are low; therefore, SRLV depends on cell-to-cell spread, allowing slow progression of infection through the monocytic lineage and inciting chronic inflammation in affected tissues [33,50].

When disease develops, lesions may involve the lungs, central nervous system, joints, or mammary gland, but the predominant clinical syndrome varies according to host species, age, viral strain, and management conditions [1,41,45]. Therefore, SRLV tropism is linked to viral genome heterogeneity and host genetics and incompletely understood host–pathogen interactions [33]. One study suggests that sequence variation in the LTR promoter/enhancer may alter interactions with cellular factors and alter viral gene expression and replication, which may affect significant changes in tissue tropism and disease progression [51]. One classic example is a duplicated motif sequence in the viral LTR that encompasses the CAAAT nucleotide sequence, leading to neurovirulence [52]. Amongst neurovirulent MVV strains are K1514 and K1772, descendants of the virus isolated from epizootic Icelandic cases between 1933 and 1965 [52,53]. Studies also suggest that LTR alone strongly promotes infection, without requiring other viral products [54]. Blatti-Cardinaux et al. [55] investigated Swiss goat flocks and observed that, despite LTR mutations that were expected to result in reduced pathogenicity, affected animals still developed histopathological lesions and exhibited high viral loads in the mammary gland.

In vitro experiments have shown that caprine cells are more susceptible to CAEV infection than ovine cells and that the virus replicates more efficiently in caprine cells, particularly in macrophages [56]. However, the viral load associated with SRLV is higher in sheep [57]. Receptors for CAEV (i.e., SRLV receptor A) and some MVV strains (i.e., SRLV receptor B) show more restricted patterns of expression, suggesting that tropism restrictions for some SRLV strains are affected by receptor expression [58]. Additionally, it is unknown whether SRLV uses only one major cellular receptor or whether receptor/co-receptor interactions are needed for viral entry and establishment of pathogenesis [33].

Certain haplotypes of the encoding ovine transmembrane protein gene 154 (TMEM154) are associated with susceptibility to MVV [59,60,61]. Specifically, sheep homozygous for a single nucleotide polymorphism (haplotype I) (rs408593969, c.103G>A, missense mutation E35K) exhibit reduced susceptibility to small ruminant lentivirus infection and may also have lower proviral concentrations and lesion severity among infected sheep, being therefore less likely to spread the infection to other sheep [62,63]. Sheep with at least one copy of either haplotype 2 or haplotype 3 were 69 times more susceptible [59,61,63]. Although the exact function of TMEM154 is unknown, the ancestral TMEM154 haplotype in sheep is common and is associated with a 191-amino-acid precursor protein that is cleaved to a mature protein with 161 residues [59,60]. A study by White et al. [64] observed that a deletion in the ovine Chemokine (C-C motif) Receptor 5 (CCR5) gene was associated with a reduced proviral load in Rambouillet, Polypay, and Columbia breeds. Additionally, Herrmann-Hoesing et al. [65] detected breed disparities in seroprevalence and provirus levels. In this study, Rambouillet sheep had lower values in both characteristics than Columbia and Polypay, suggesting mechanisms that prevent infection and/or aid virus control after infection [57].

MVV and CAEV use different cell receptors to enter host cells, including the mannose receptor (MR, encoded in mice on chromosome 2), which appears to be a major receptor in sheep [22,33,66]. Three cell phenotypes have been hypothesized in small ruminants in relation to the expression and usage of MR as an SRLV receptor: cells employing this molecule as the main receptor (synovial membrane cells), cells having this and at least another main SRLV receptor(s) (macrophages), and cells lacking MR and using other SRLV receptor(s) instead (skin fibroblasts) [66]. Additional research also suggested that MR presence was associated with disease progression, and that increased MR expression was related to severe disease in target organs [67].

Toll-like receptors (TLRs) are host pattern recognition receptors that have an essential function in the innate immune system and are activated during SRLV infection [33,41]. TLRs recognize a variety of conserved pathogen-associated molecular patterns and induce intracellular signaling pathways involving a series of phosphorylation events mediated through the common adaptor molecule MyD88 [68]. These signaling pathways activate and translocate NF-kB or AP1 transcription factors to the nucleus, leading to pro-inflammatory cytokine production and recruitment of innate effector cells to clear the infection [69]. MyD88 recruitment and activation of either NF-kB or AP1 are conserved across TLRs 1, 2, 4, 5, 6, 7, 8, and 9 [68]. Many TLRs, including *TLR2*, *TLR4*, *TLR7*, and *TLR8*, were upregulated in the lungs of SRLV-infected animals compared with uninfected animals, suggesting MyD88 use and the previously described signaling pathway [70,71,72]. These TLRs, especially *TLR7* and *TLR8*, once activated, promote IFN-gamma, IL-6, and TNF-alpha production and subsequent antiviral protein expression [33]. Additionally, some polymorphisms in the *TLR7* and *TLR8* genes were linked to SRLV proviral load in goats and sheep, suggesting these genes may trigger a differential innate immune response to SRLV that affects proviral load and, ultimately, disease pathogenesis and progression [73,74]. It remains unclear whether TLR signaling pathways induce SRLV replication or whether the SRLV genome has an NF-κB transcriptional binding site in the promoter [50].

SRLV infection induces both innate and adaptive immune responses [33]. Once the virus enters the target cell, molecules of the innate and adaptive immune systems start host restriction. The best-known viral antigens triggering specific adaptive immune responses are the ENV and GAG proteins [1]. Antigen-presenting cells such as monocytes and macrophages process and present these viral antigens to the immune system [1]. Immune activation of macrophages increases SRLV replication, including stimulation of MAPK and protein kinase C, which are directly linked to expression from the viral LTR [33,75,76]. Macrophages and dendritic cells act as antigen-presenting cells that stimulate T-cell responses, thereby activating specific cytotoxic T cells, promoting helper T-cell (Th) differentiation, and driving B-cell-derived antibody production [1,33,50]. MAPK and protein kinase C signaling is stimulated by cytokines such as GM-CSF, IL-1beta, IFN-gamma, and TNF-alpha [33,45,77,78]. The adaptive immune response is highly antigen-specific and dependent on small ruminant lentivirus heterogeneity [1].

Innate immunity molecules, including tripartite motif-containing 5 (TRIM5), bind viral capsids, inhibiting integration and post-integration steps of the viral cycle, while others, such as Apolipoprotein B mRNA-editing catalytic polypeptide-like 3 (APOBEC3 or A3), mutate the viral genome [23,43,44,45]. Type I interferon (IFN) is one of the most important mediators of innate immunity against viral infections, including retroviruses, which are considered weak inducers of type I IFN [33,45]. IFNs bind to specific receptors on the cell membrane, such as IFNAR1 and IFNAR2, to trigger the antiviral response, leading to a cascade of signal pathways that aim to reject the viral infection by the cell [79]. Type I IFN can also interfere with monocyte proliferation and differentiation [33]. In small ruminants, three types of IFNs have been identified: type-I (IFN-I), which includes IFN-α, IFNβ, and many others (IFN-ω and IFN-τ), produced by most virus-infected cells; type-II (IFN-II), which includes only IFN-γ and is produced only by certain cells of the immune system; and type-III (IFN-III), including IFN-λ, which were formerly interleukins [43]. In an experimental study, lambs treated with type I IFN immediately after MVV infection had decreased viral load and reduced disease development [80]. Apobec3G, TRIM5alpha, and Tetherin (*BST-2*), also called intrinsic resistance mediators that may be related to lower proviral loads and possible protection against viral shedding and disease initiation, are up-regulated by type I IFN treatment [33,41].

The major histocompatibility complex (MHC) region, a polymorphic multi-gene complex located on chromosome 20 in sheep and chromosome 23 in goats, has been implicated in SRLV infection and SRLV-induced disease [81,82]. In sheep, MVV infection increases MHC Class II mRNA expression in the lung, CNS, and synovium [82]. CAE virus-induced arthritis in goats is genetically influenced by the MHC system, and susceptibility/resistance genes are not directly associated with the determined class I gene products but rather are in close genetic linkage [83]. Ruff et al. [84] proposed that protection from CAEV-induced arthritis is linked to expression of the caprine leucocyte antigen MHC class I allele; however, the particular class I CLA allele associated with the disease susceptibility varied from family to family. Additionally, this study demonstrated that CLA Be7 specificity in the Saanen caprine breed is linked to protection from CAEV-induced arthritis [83,84].

The cell-mediated immune responses against SRLVs begin 1 to 4 weeks post-infection; however, antibody responses can emerge as early as 2 to 4 weeks post-infection and tend to fluctuate during the first 6 months of infection [43,85]. Proliferative and cytotoxic T cell responses, including CD4+ and CD8+ T cell responses, are induced after SRLV infection; however, the virus is not cleared, and inflammation persists throughout the infection [33,45,50]. Although SRLV does not target CD4+ cells, unlike other lentiviruses, it is hypothesized that CD4+ cells play an important role in establishing macrophage infection, but not dendritic cell infection [47]. Stonos et al. [50] suggest that SRLV infection interferes with antigen processing and presentation, likely limiting the ability of antigen-presenting cells to activate CD4+ T cells and induce cytotoxic T lymphocyte responses.

Additionally, T cell proliferative response decreases in late-stage disease following decreased antigen-presenting function, with reduced CD80 expression and no reduction in CD86 expression [79]. In a study, authors reported that CD80 and CD86 RNA levels were associated with the capability of peripheral blood mononuclear cells to respond to MVV *gag* antigens, including p14, p17, and p25, by proliferation, which was demonstrated with most seropositive asymptomatic sheep showing positive proliferative responses but clinically affected sheep showing no response, suggesting possible anergy which impedes defense against the virus, blocking the mount of memory responses [43,86]. Therefore, among seropositive sheep, decreased CD80 RNA levels and decreased antigen-specific cellular immune responses in peripheral blood mononuclear cells imply MVV disease status, whereas increased CD80 and CD86 levels and intensified cellular responses are linked to asymptomatic infection [86].

In ruminants, γδT cells comprise approximately 70% of all lymphocytes in young animals and play a major role in the innate immune system [87,88]. Studies have demonstrated that CAEV-infected goats have a significantly higher proportion of γδT cells than healthy goats, suggesting these cells may help control SRLV infection by limiting SRLV entry and mediating early immune responses [87,88].

SLRV can alter macrophage function and maturation, leading to immune dysfunction and modulation of viral replication [1,44]. To investigate this immune dysfunction, Lechner and collaborators [89] examined cytokine profiles of CAEV-infected macrophages in culture [89,90]. Observations from these studies included that infected macrophages’ TGF-β mRNA were down-regulated while producing abundant IL-8 and monocyte chemotactic protein-1 (MCP1) [89,90]. In lipopolysaccharide (LPS)-stimulated CAEV-infected macrophages, the authors also reported increased GM-CSF mRNA expression and decreased TNF-α, IL-1β, IL-6, and IL-12 mRNA expression [89,90]. These findings suggest that CAEV infection may dysregulate cytokine expression in macrophages and possibly modulate the accessory functions of infected macrophages and the antiviral immune response in vivo [89]. Increased levels of GM-CSF were also identified in alveolar macrophages of MVV-infected sheep, suggesting that the infection can induce a phenotypic shift in macrophages from pro-inflammatory M1 to anti-inflammatory M2 macrophages that favors viral replication [77,91]. This shift to anti-inflammatory M2 macrophages is also described in outbreaks of the neurologic form of MVV [92].

Systemic responses may take several months to be detectable after natural infection, but immune responses in the lymphatic system draining a site of experimental infection are detectable within 4 days [48]. Studies have demonstrated compartmentalization of immune responses within affected organs [33,45,50]. In goats with arthritis, certain joint regions may exhibit high concentrations of IFN-gamma and Th1-type responses, whereas other areas of the same joint have an inflammatory infiltrate composed of plasma cells, suggesting Th2-dominated responses [90]. This may explain differences in inflammatory infiltrates that can arise from different organs [33,50].

In the humoral response, the first antibodies may appear 3 weeks after experimental infection and target p25CA. Approximately 45 days after infection, sheep and goats synthesize antibodies against gp46TM, p14NC, and p16MA, and later generate antibodies against gp135SU [93]. The antibody response to SRLV generally targets epitopes on the gp135, gp38, and capsid proteins [94]. After natural SRLV infection, animals produce IgM and IgG antibodies [33,45]. In goats, an IgG1-dominated response is induced [95]; however, only IgG1 and no IgM anti-MVV responses are detected in sheep [96]. The dominant production of IgG1 in ruminants is suggestive of a type 2 T helper cell (Th2) with a dominance of IL-4 production, meaning IgG1 expression is positively regulated by IL-4 in SRLV-infected animals [97]. For example, in goats infected with CAEV, those that develop a strong IgG1 antibody response (Th2-biased CD4+T cell response) develop arthritis, while those with a mixed IgG1 and IgG2 response usually remain subclinical, suggesting that anti-SRLV IgG2 antibodies protect against clinical disease [95,98].

Other lentiviruses of importance in veterinary and human medicine (Table 1) include human immunodeficiency virus (HIV), simian immunodeficiency virus (SIV) in primates, feline immunodeficiency virus (FIV) in cats, and equine infectious anemia virus (EIAV) in horses. The members of the lentivirus genus differ in disease development and cellular tropism [34]. Viruses such as HIV, FIV, and SIV replicate in both macrophages and lymphocytes, inducing apoptosis in infected and uninfected CD4+ T lymphocytes and depleting these cells, a characteristic of immunodeficiency caused by these viruses [21,34]. Both SRLV and EIAV replicate primarily in macrophages and do not infect lymphocytes [34]. Therefore, contrary to what happens in feline and primate lentiviruses, SRLVs do not induce immunodeficiency in goats and sheep, as the virus does not target CD4+ T lymphocytes but rather induces a chronic inflammatory response [21,45]. Additionally, in SRLV-infected animals, immunosuppression reduces both lesions and viral load [99].

## 3. Clinicopathologic Findings

SRLVs cause a progressive multisystemic inflammatory disease in which the central change is infiltration of several tissues by mononuclear cells, particularly macrophages, lymphocyte subsets, and plasma cells [33]. About 30% of animals infected with the virus develop some clinical signs, while others may remain subclinical [2]. The disease is insidious, and clinical signs are generally seen in adult animals, mainly sheep, over two years old [1,33]. Additionally, infected young goats are more prone to develop leukoencephalitis, while adults frequently develop chronic arthritis [100,101]. Clinical signs appear to depend on the tropism of the small ruminant lentivirus strain, the species affected, and the genetic background of each breed or animal [1]. The four major tissues affected by SRLV-induced disease in both sheep and goats are the lung, CNS, mammary gland, and joints [1,102,103]. In sheep, respiratory and mammary forms predominate, whereas in goats, the joints and central nervous system are more commonly affected [33,43]. However, inflammation and tissue damage can also be seen in other organs, including the kidney, liver, and heart [1,101,104]. Details of each disease form are discussed below.

Table 2 includes differential diagnoses for all forms of the disease.

### 3.1. Lungs

Historically, the respiratory form has been the most frequent and the easiest to recognize. Luján et al. [102] report that the respiratory form of SRLV in sheep dates back to the 19th century in the Netherlands, shortly followed by descriptions in South Africa (“Graaff-Reinet disease”), France (“La Bouite”), and the United States of America (“Montana disease”). In Iceland, in 1933, 20 Karakul lambs infected with SRLV were imported from Germany, quarantined, and distributed to selected farms [53]. This sheep import was considered the most disastrous import in the history of mankind, because not only were these lambs infected with SRLV, but they were also infected with Jaagsiekte sheep retrovirus (ovine pulmonary adenocarcinoma—OPA) and Johne’s disease (paratuberculosis), leading to more than half a million sheep deaths and almost complete disappearance of the local Icelandic breed [53]. The respiratory form of SRLV corresponds to maedi, which means “labored breathing” in Icelandic [53].

The respiratory form is the most common presentation of small ruminant lentivirus in sheep, mainly in adults (over 2 years old), and has also been reported in growing animals (younger than 1-year-old lambs) [93,105]. Once sheep begin exhibiting respiratory distress, life expectancy does not exceed one year [105]. The respiratory form is described to be the most relevant with regard to prevalence and financial significance in sheep, whereas it seems infrequent in domestic goats [1]. Clinical signs may vary with disease progression and stage of development [102]. Clinically, affected animals exhibit weakness, increased respiratory rate, abdominal breathing, neck extension, nostril expansion, mouth-open breathing, weight loss, and exercise intolerance [7,102,106,107]. Fever, cough, or nasal discharge are not common clinical signs of SRLV infection in sheep; however, they may be present in cases complicated by secondary bacterial infections or coinfections with Jaagsiekte sheep retrovirus [43,102].

Macroscopically, the lungs fail to collapse, are heavy and increased in volume, and exhibit homogeneous grey spots throughout the serosal surface [7,102,108]. Bronchial and mediastinal lymph nodes are enlarged and edematous, surpassing the limit of the diaphragmatic lobes [45,102]. Edema is uncommon unless associated with a secondary infection [107].

The main histologic lesion is lymphocytic interstitial pneumonia, characterized by secondary lymphoid nodules around vessels and airways with a predominance of CD8+ T cells [102]. Additionally, alveolar septa thicken extensively due to smooth muscle hypertrophy [1,7,102,108,109]. Type II pneumocyte hyperplasia may be observed, but less commonly than smooth muscle hypertrophy [1,7,108]. These pulmonary changes reflect ongoing lymphoid recruitment within the interstitium, along with smooth muscle hypertrophy and mild interstitial fibrosis, due to ongoing activation and growth-factor release by macrophages and lymphocytes [7,11,102]. Severe perivascular fibrosis can be observed in large pulmonary arteries [45]. In cases with enlarged mediastinal lymph nodes, histological lesions are consistent with cortical hyperplasia [45]. Immunohistochemistry and in situ hybridization can localize the virus within type I and II pneumocytes, interstitial and alveolar macrophages, endothelial cells, and fibroblast-like cells in the pulmonary parenchyma [44,110].

In sheep, MVV is often associated with concurrent diseases, including ovine pulmonary adenocarcinoma (OPA) caused by Jaagsiekte sheep retrovirus, bacterial bronchopneumonia such as *Pasteurella multocida* and *Mannheimia haemolytica*, and parasites (i.e., *Muelleria capillaris*), which may interfere with MVV diagnosis [102,111,112,113]. Analysis of cases of coinfection with Jaagsiekte sheep retrovirus and MVV suggests that MVV infection possibly exacerbates persistent inflammatory processes in the lungs, contributing to the development of pulmonary adenocarcinoma [113].

Chronic interstitial pneumonia in goats infected with SRLVs is much less common than in sheep; however, similar macroscopic and histological lesions have been described, including perivascular lymphoid cuffing, type II pneumocyte hyperplasia, and thickening of alveolar septa [114]. In a large study performed in Poland, in a goat farm extensively affected by CAEV, caused by SRLV genotype A, researchers noticed that between 50 and 60% of infected goats had histological lesions compatible with interstitial pneumonia, and these lesions were often associated with other lung diseases such as bacterial infections [100]. In a study on dairy goat farms, animals infected with CAEV had a higher frequency of caseous lymphadenitis (*Corynebacterium pseudotuberculosis* infection) than non-infected animals, suggesting a positive, albeit low-intensity, association [115].

### 3.2. Central Nervous System

The neurologic form of the disease in sheep is sporadic and is called visna, which means “wasting” in Icelandic. This form was first reported in Iceland in 1957 [116], and sporadic cases have since been described worldwide [7,9,92,103]. In contrast to the respiratory form, which affects only adults, the neurological form of SRLVs has been described in sheep as young as 4 months old, although mature animals (over 2 years old) are more commonly affected [7,92]. Common clinical signs include ataxia, hindlimb paralysis that can progress to paralysis in four limbs, hypermetria, and blindness [2,7]. In cases in which the lesions are concentrated in the spinal cord, animals may exhibit recumbency and knuckling of 1 of the rear limb fetlocks even while the limb stays weight-bearing [43,103].

In young goats, usually between 1 and 6 months old, infected with CAEV, the clinical course is characterized by an afebrile disease with unilateral or bilateral posterior paresis, ataxia, and ascending paralysis due to acute leucoencephalomyelitis [117]. In the early stages, the animal may demonstrate a short, choppy gait. Cranial signs, including head tilt and circling, may be observed. Affected animals are typically alert and responsive and continue to nurse, eat, and drink normally [92,103]. Posterior paresis progresses to tetraparesis over a period of several weeks [118]. CAE with nervous signs is characterized by acute demyelinating leucoencephalomyelitis with neuronal damage [118,119]. However, chronic demyelinating leucoencephalomyelitis with severe demyelinated foci, resembling multiple sclerosis, with marked astrocytosis and inflammatory response is also common in adult animals [118,119]. Authors have described that detectable viral antigen in CAE decreases with increasing inflammation associated with disease duration, suggesting that antibody-independent cytotoxicity may be involved in the pathogenesis of CAE demyelinating leucoencephalomyelitis in the acute phases, whereas antibody-dependent T-cell cytotoxicity and delayed-type immune-mediated mechanisms are implicated in the chronic stages [119].

Histologically, neurological SRLVs are characterized by non-suppurative encephalomyelitis with perivascular and periventricular inflammation composed of lymphocytes, plasma cells, and histiocytes, as well as areas of malacia, demyelination, choroiditis, and meningitis [7,11,103]. The infiltration of the choroid plexus by lymphocytes may lead to the formation of ectopic lymphoid follicles [120]. As in the brain, spinal cord lesions are also associated with histiocytic infiltrate targeting the white matter [92].

Benavides et al. [103] described the lesions in three patterns: a vascular pattern characterized by mononuclear cells surrounding blood vessels (perivascular cuffs); an infiltrative pattern with mononuclear infiltration of the neuroparenchyma in addition to perivascular cuffing; and a malacic pattern with demyelination. Additional research further classified the lesions as dependent on the main cellular composition and presence of viral antigen in two different patterns: a lymphocytic pattern characterized by an infiltrate with CD8+ T lymphocytes with scarce viral antigen; and a histiocytic type with extensive areas of malacia associated with numerous macrophages (CD163+/M2 macrophage), B cells, and abundant viral antigen presence [121]. These findings are in accordance with the previously discussed immunopathogenesis of SRLV, in which faulty macrophages are not able to control viral replication, thus leading to the presence of abundant viral antigen, while the presence of CD8+ T lymphocytes can control but not completely clear the infection, leading to persistent infiltration of these cells in tissue with the presence of some viral antigen [121].

A study of naturally SRLV-infected goat kids between 40 and 60 days of age with clinical paresis and paralysis, involuntary muscular tremors, and occasional torticollis demonstrated that acute demyelinating leucoencephalomyelitis was the most conspicuous microscopic change in the brain and spinal cord [119]. Additionally, this study discussed the important role of apoptosis in the pathogenesis of CAE demyelinating encephalitis by showing labeling for anti-bax and anti-caspases-3, -8, and -9 in a distinct population of glial cells, mainly astrocytes and a few oligodendroglia [119].

### 3.3. Joints

As previously mentioned, arthritis is the least common presentation of MVV but is frequently observed in CAEV-infected goats [10,43,109]. This lesion usually affects animals between 2 and 3 years old [43]. In both sheep and goats, the articular form involves the presence of enlarged joints, mainly affecting the carpal but also the tarsal joint and, rarely, other locations, such as the atlantal bursa and the nuchal ligament [21,34,49]. One or many joints can be affected at the same time [2]. The lesion initially presents with edema and congestion of the joint capsule, leading to enlargement of the affected joint [10]. Macroscopically, the lesions consist of hardening of the periarticular tissue, with progressive mineralization, apparent synovial membrane proliferation, and multifocal erosions of the articular surfaces [10]. Additional lesions, depending on chronicity, include bony exostoses, joint collapse, and eventual ankylosis [2]. The decreased quantity of articular fluid is also described [10].

Histologically, joint lesions associated with SRLV infection include papillary synoviocyte proliferation with synovial membrane thickening due to fibrosis, mineralization, and multifocal lymphocytic, plasmacytic, and histiocytic inflammation [45]. Clusters of plasma cells and binucleated or multinucleated syncytia may be observed in the connective tissue of the proliferated synovial membrane [10,122]. Multifocally, villous hypertrophy, angiogenesis, and synovial membrane necrosis are common [33,44]. In more severe cases, cartilage and bone destruction can be observed and usually consists of necrosis with dystrophic calcification and cartilaginous and/or osseous metaplasia [10,122]. In some cases, degeneration of the arterial tunica media and thrombi within the blood vessels of affected joints were noted [10]. Immunohistochemistry reveals a high number of macrophages and CD8+ T cells in affected joints, with an increased proportion of B cells in lesions as infection progresses [123]. Immunocytochemistry can identify SRLV-infected cells in the articular fluid [10]. Studies differ on the number of SRLV-infected cells in the articular fluid, and the hypothesis is that viral phenotype and strain play a major role in whether these cells are present [10,122].

Wilkerson et al. [124] while investigating the immunopathology of chronic lentivirus-induced arthritis in Saanen goats, demonstrated the presence of CD68+ T cells lining the surface of the inflamed villi and reported CD45R+ CD5- B lymphocytes as the principal component of most perivascular infiltrates in arthritic synovium. Inflamed villi also contained lymphoid aggregates comprising B cell germinal centers and activated T-cell mantles [124]. This investigation concluded that memory immune responses that favor B-cell expansion and maturation and immunoglobulin production contribute to the immunopathology of chronic arthritis in goats [124]. Another immunohistochemical study in Rasa Aragonesa sheep experimentally infected with an arthritis-related strain [10,125] reported that CD68+ cells were more abundant in cases with severe arthritis [45].

In Spain, Pérez et al. [10] described clinicopathologic features of an arthritis outbreak in sheep induced by a new SRLV isolate phylogenetically assigned to caprine arthritis encephalitis virus-like subgroup B2. This study discussed that the arthritis component in sheep may be underestimated, since the virus was observed in cells in the subsynovial connective tissue even in animals with no signs of locomotor problems [10]. Many animals develop progressive weight loss due to decreased range of motion and constant pain from the lesions [2]. Due to its refractory nature to treatment, it frequently leads to euthanasia due to deterioration of the animal’s clinical health [2].

Recently, researchers applied ozone therapy to treat different severities of arthritis in naturally infected SRLV goats [126]. The results of this study suggest that ozone therapy can be utilized to improve clinical signs and production rates in dairy SRLV goats with chronic arthritis and weight loss [126].

### 3.4. Mammary Gland

The mammary syndrome can affect both sheep and goats and has important financial and epidemiological consequences [1]. Lujan et al. [127] discussed that in some breeds as the Spanish breed Rasa Aragonesa, the mammary form may be more frequent than the respiratory form. Affected animals range in age from 3 to 5 years, but lesions have been observed in animals as young as 1 year [38,118]. Studies have shown that this form of SRLV is more frequent in mechanically milked and intensively reared animals [22].

SRLV-associated mastitis is challenging to detect. Macroscopically, it is characterized by udder hardening and reduced milk production, both of which tend to occur after parturition [128]. Both adult does and ewes can develop bilateral indurative mastitis [2]. Mastitis with swollen mammary lymph nodes is typically subclinical, indurative, recurrent, diffuse, bilateral, and nonpainful [129].

Mammary gland lesions are characterized by periacinar lymphocytic interstitial mastitis and lymphoid hyperplasia, with a predominance of CD3+ T cells and fewer histiocytes [11]. Bolea et al. [128] demonstrated replication of small ruminant lentiviruses in macrophages or acinar epithelial cells, which induces the prominent lymphocytic component of the mastitis. SRLV-infected epithelial cells, macrophages, endothelial cells, and fibroblast-like cells in the mammary gland can be visualized by in situ PCR-associated immunohistochemistry [110]. Additional lesions include spread fibrosis leading to stenosis, with areas of calcification and gland atrophy, complete loss of acini, ductal epithelial hyperplasia, smooth muscle hyperplasia, vacuolization, and desquamation [129]. Hyperplastic lymphoid follicles can be observed throughout the parenchymal tissue in the acini and around lactiferous ducts [1,2].

Mastitis may also reduce milk production, thereby reducing milk availability to lambs and affecting lamb growth [129,130]. Interestingly, the reduced milk production caused by SRLV infection is not only a direct result of acinar loss in the mammary gland but is also related to a decreased metabolic rate due to systemic disease [130,131].

Various reports describe the presence or absence of changes in milk properties in infected goats. In some reports, authors describe no consistent changes in milk [122]. Others report higher pH, chloride content, electrical conductivity, and somatic cell counts [129,132,133]. In two studies, authors reported an approximately 15% decrease in milk production, associated with decreased fat content and lower milk quality [132,134].

## 4. Current Limitations of Lentiviral Diagnosis

In sheep and goats, SRLV infection is often prolonged and clinically inapparent, allowing infected animals to remain as reservoirs within flocks and herds [1,135]. As a result, clinical signs and postmortem findings alone are insufficient for accurate diagnosis or surveillance [1,34,136,137,138]. Diagnostic confidence is greatest when clinical and postmortem findings are interpreted in combination with serology, molecular testing, histopathology, and, where available, detection of viral antigen or proviral DNA in affected tissues [1,41,45].

Postmortem examinations can support the diagnosis of SRLV infection through macroscopic evaluation, histopathology, and tissue-based detection of viral antigen or proviral DNA [10,11,44,103]. Diagnostically relevant changes may be missed if tissue sampling is incomplete or not targeted to the affected organ system [1,10,11,44,45,92]. Thus, postmortem interpretation depends on integrating lesion distribution, appropriate tissue selection, sample preservation, and the diagnostic performance of ancillary methods [10,11,44,92,139].

Future diagnostic improvements require well-defined, validated diagnostic panels that capture viral diversity across distinct host species, geographic regions, sample types, and infection stages [23,41,135,139]. Additionally, standardized diagnostic algorithms supported by reference laboratories are needed to guide the appropriate use of serology, molecular testing, and tissue-based methods, as well as the interpretation of discordant results [23,41,135,138,139,140]. These strategies would improve interlaboratory comparisons, reduce discrepancies in regional test interpretation, and strengthen SRLV control efforts [138,139,140]. Until these limitations are addressed, false-negative results, assay discrepancies, and inconsistent classification of infected flocks and herds will remain major obstacles to diagnosing and controlling SRLV infection [34,41,137,139,141].

## 5. Conclusions

Small ruminant lentivirus is an important chronic disease present in many major sheep and goat production systems. SRLV tropism for the monocytic–macrophagic lineage is linked to viral genome heterogeneity, host genetics, and host–pathogen interactions, and many cellular receptors are still unknown. Future studies on the specific genes involved in susceptibility or resistance to SRLV infection are warranted. Additionally, investigations into how different SRLV strains affect macrophage function, cytokine production, and intracellular signaling should be conducted to better clarify SRLV immunopathogenesis.

## Figures and Tables

**Table 1:** Lentiviruses of importance in veterinary and human medicine.

Disease Name	Virus	Main Hosts	Main Target Cells
HIV infection/AIDS	HIV-1 (Human immunodeficiency virus 1)	Humans	CD4+ T helper lymphocytes
HIV infection/AIDS	HIV-2 (Human immunodeficiency virus 2)	Humans	CD4+ T helper lymphocytes
Simian immunodeficiency virus infection (simian AIDS-like disease)	SIV (Simian Immunodeficiency virus)	Non-human primates	CD4+ T helper lymphocytes
Feline immunodeficiency	FIV (Feline immunodeficiency virus)	Felids	CD4+ T cells, CD8+ T cells
Equine infectious anemia	EIAV (equine infectious anemia virus)	Equids	Macrophages and monocytes
Bovine immunodeficiency virus infection	BIV (Bovine immunodeficiency virus)	Cattle	Monocytes, macrophages and lymphocytes

**Table 2:** Pathological findings and differential diagnosis of small ruminant lentiviruses.

Presentation	Key Presentation Features	Main Differential Diagnoses
Respiratory (Maedi)	Heavy lungs that fail to collapse, chronic lymphocytic interstitial pneumonia with lymphoid follicles around airways and vessels, smooth muscle hyperplasia	OPA, chronic bacterial pneumonia, lungworm (can also cause smooth muscle hyperplasia) *Mycoplasma ovipneumoniae*
Central nervous system (Visna)	Non-suppurative encephalomyelitis or leukoencephalitis with perivascular cuffing	Listeriosis, polyencephalomalacia, scrapie
Joints	Proliferative nonsuppurative arthritis	Degenerative joint diseases, traumatic arthritis, chronic arthritis
Multisystemic	Progressive weight loss associated with chronic involvement of multiple organs with a lymphoplasmacytic and/or histiocytic infiltrate	Parasitism, Johne’s disease
Mammary gland	Lymphocytic interstitial mastitis	Mycoplasma mastitis, chronic bacterial mastitis

## Data Availability

No new data was created in this study.

## Abbreviations

The following abbreviations are used in this manuscript:

SRLV	Small Ruminant Lentiviruses
MVV	Maedi-Visna Virus
CAE	Caprine Arthritis-Encephalitis Virus
